# A Narrative Review of the Current State of Extended Reality Technology and How it can be Utilised in Sport

**DOI:** 10.1007/s40279-022-01669-0

**Published:** 2022-03-14

**Authors:** Peter Le Noury, Remco Polman, Michael Maloney, Adam Gorman

**Affiliations:** 1 Faculty of Health, School of Exercise and Nutrition Sciences, Queensland University of Technology, 502/510 Saint Pauls Terrace, Bowen Hills, Brisbane, QLD 4006 Australia; 2 Australian Institute of Sport, Canberra, ACT Australia

## Abstract

Extended reality is an umbrella term used to describe three computer-generated technologies including virtual reality, augmented reality and mixed reality. Extended reality is an emerging technology that has been utilised in many high-performance domains including psychology, medicine and the military, with the aim of enhancing perceptual-cognitive skills and motor skills. However, the use of extended reality in sport, particularly at the elite level, has only recently started to receive attention. While the growth of extended reality technology continues to accelerate at a rapid rate, empirical evidence aimed at understanding how these devices can best be applied in high-performance sport has not followed suit. Therefore, the purpose of this review is to provide clarity for high-performance sport organisations, researchers, sport scientists, coaches and athletes about the current state of extended reality technology and how it has been utilised in sport. In doing so, we first define and give examples of the types of extended reality technology including virtual reality, augmented reality and mixed reality that are available at the present time. Second, we detail how skill acquisition principles underpinned by the theoretical framework of ecological dynamics can be used to help inform the design and assessment of extended reality training tools. Third, we describe how extended reality has been utilised in sport, including how extended reality tools have been assessed for their level of representativeness, and the effectiveness of extended reality training interventions for improving perceptual-cognitive skills and motor skills. Finally, we discuss the future utilisation of extended reality in sport, including the key learnings that can be drawn from other domains, future research directions, practical applications and areas for consideration related to the use of extended reality for training skills in sport.

## Key Points

**Table:** 

It is important to base the design of sport-specific extended reality (XR) tools on the key principles of ecological dynamics and representative learning design, and utilise the modified perceptual training framework to ensure that XR tools are highly representative of the real-world performance environment to maximise positive transfer.
To validate the use of XR tools and minimise the probability of negative transfer effects, it is essential that XR tools are assessed for their level of representativeness before they are used during training.
As interest in XR technology grows throughout the high-performance sport landscape, it is important to maintain a balanced and evidence-based approach when deciding how XR can best be utilised within training programmes.

## Introduction

Perceptual-motor skills are an essential part of skilled performance in sport [1]. Finding novel ways to train these types of skills has long been a strong interest of coaches and managers within high-performance programmes with the aim being to gain a competitive advantage. Extended reality (XR), which is an umbrella term that encapsulates all real and virtual environments that are generated by computer technology and wearables [2], provides an opportunity to potentially fast track the development of such skills. Extended reality technologies have been utilised in high-performance environments across multiple domains such as psychology [3], medicine [4] and the military [5]. The appeal of XR within these contexts is that it can re-create environments that are challenging to simulate in training and provide a safer training environment with reduced risk of injury and/or damage to expensive equipment. Additional advantages of XR include the capability to control and manipulate constraints in complex and dynamic environments to create specific situations that are repeatable (e.g. landing an airplane after an engine failure during a flight simulation). Extended reality has also been utilised in sport, albeit to a lesser degree compared with other domains, with high-performance programmes around the world only recently adopting this technology and investigating its efficacy for improving athlete performance. However, the adoption of XR in the sporting domain is outpacing the generation of scientific research evidence, leading to a gap between the practical application of XR technology and the empirical understanding of the extent to which it is able to improve perceptual-motor skills in sport.

Two key issues preventing high-performance sporting organisations from investing the time and money required to develop XR training tools are the lack of understanding about (1) XR tools (i.e. what is possible from a hardware and software perspective) and (2) the utility of XR tools to elicit positive skill transfer. With respect to the first issue, greater clarity is required around the definition of XR and exactly what it entails. Moreover, the sports domain can learn from the information generated from XR research in other domains (e.g. psychology and military). Preliminary research investigating the efficacy of sport-specific XR tools, including 360-degree virtual reality (VR), animated VR, and augmented reality (AR), for skill assessment and training can also provide valuable insights into the future utilisation of XR in sport [6]. With respect to the second issue, a principled approach (informed by ecological dynamics) to technology and task design assessment can help inform stakeholders about the efficacy of various XR technologies. Ecological dynamics is a principled theoretical approach that can provide guidance to practitioners aiming to understand and assess performance in sport. The ecological dynamics approach focuses on the individual-environment relationship to analyse and understand performance [7]. Through an ecological dynamics lens, athlete behaviour is thought to be shaped by the continuous interactions between task (e.g. rules and equipment), environmental (weather conditions, lighting, socio-cultural) and individual (e.g. physical and psychological characteristics) constraints that exist within the performance environment at any given time [7]. Considering these constraints and the dynamic relationship between the performer and the environment (i.e. the information available and the way the individuals interact with that information) within a training setting, is essential for understanding whether skills will transfer across environments, such as from XR to competition [8, 9]. These ideas have been operationalised as applied frameworks through representative learning design (RLD) [9] and the modified perceptual training framework (MPTF) [10]. These frameworks can be used by practitioners to inform the design of skill learning tasks when using XR technology and can help assess the probability of the learning task eliciting positive skill transfer to the performance setting [9–11].

This review aims to provide clarity for high-performance sport organisations and the broader sports research community about the current state of XR technology and its efficacy to be utilised in sport. The first section of this review provides definitions and examples of the types of XR technology including VR, AR and mixed reality (MR) that are available at the present time. The second section details how skill acquisition principles including ecological dynamics, RLD, and the MPTF can be used to help inform the design of XR training tools. The third section describes how XR has been utilised in sport, including how XR tools have been assessed for their level of representativeness (i.e. the extent to which XR represents the real-world setting), and the effectiveness of XR training interventions for improving perceptual-cognitive and motor skills. The final section discusses the future of XR in the sporting domain by outlining how research from other domains can inform the utilisation of XR in sport, providing future research directions and practical applications for XR in sport, and explaining some areas for consideration related to the use of XR technology for enhancing perceptual-motor skills in the sporting domain.

## Methods

The articles discussed in this narrative review were first located using a combination of search terms including “virtual reality”, “augmented reality”, “mixed reality”, “artificial intelligence”, “robotics”, “sports performance”, “perceptual-cognitive skill”, “motor skill”, “psychology”, “military”, “aviation” “representativeness”, “fidelity” and “ecological dynamics” in Sport Discus, PubMed, Science Direct, Web of Science and Scopus. A literature search was also conducted using Google Scholar with the above search terms, and we used a snowballing literature search method by identifying relevant articles within the reference lists of previously published studies and reviews in the area of interest. One author then screened all titles and abstracts of the articles identified and rejected any that were clearly irrelevant. Given the narrative nature of this review, manual searches through the reference lists of included articles, and all authors personal database of references relating to the topic of interest, were also undertaken to retrieve any articles that had not been identified through the abovementioned search method. All potential articles were then read in full by one author who discussed specific aspects of articles with the other authors where necessary to reach consensus regarding their inclusion. A further search was conducted during the drafting phase of the paper to provide further detail, support or evidence relating to particular aspects of the review where required. Given the use of XR tools is in its infancy in sport, our best efforts were made to discuss, or at least make reference to, all published articles that have assessed the utility of XR in sport that we felt were relevant.

## Defining XR

**Fig. 1:**
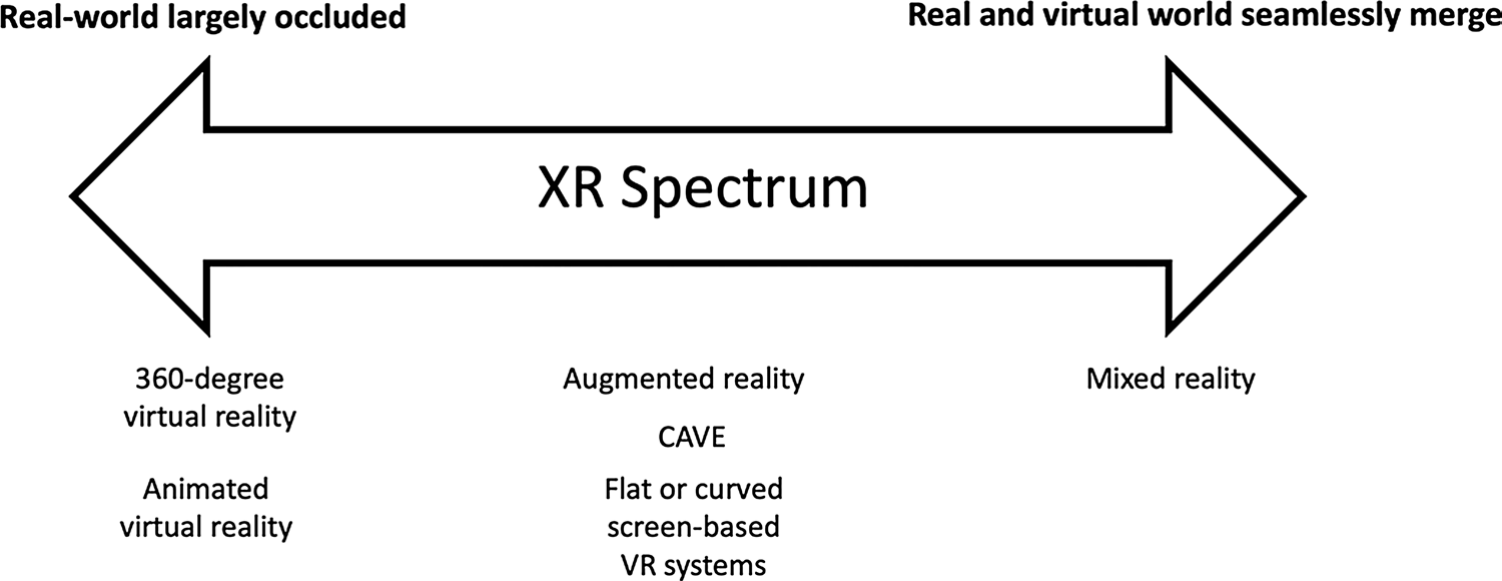
Extended reality (XR) spectrum, from tools that largely occlude the real world to tools that seamlessly combine the real and virtual worlds. *VR* virtual reality

Extended reality is the umbrella term that refers to three different types of computer-generated simulations including VR, AR and MR [2]. Although these three modalities have specific characteristics, the differences between each have become somewhat blurred in recent times (e.g. being able to interact with and influence objects in an animated VR environment; however, not in a 360-degree VR environment). This may have occurred for various reasons, including the creation of marketing terms in order to sell products, or the extremely fast rate of development of these technologies, which makes it difficult for the general public to keep up with new technology trends and developments. Therefore, we suggest the clearest way to describe XR modalities is as a *spectrum* that moves from one modality to another. This spectrum ranges from modalities that are completely virtual in nature and largely occlude the real world, to modalities at the other end of the spectrum whereby the real and virtual world seamlessly merge, are aware of each other, and can interact with one another naturally and in real time (see Fig. 1). The next section discusses the characteristics of each modality along the XR spectrum.

### Key Difference Between Animated VR and 360-Degree VR

It is important to note before describing these terms in more detail that there is a significant difference between an *animated* VR simulation and 360-degree video presented in a VR headset (commonly referred to as 360-degree VR). There has been some confusion amongst practitioners and researchers about the difference between these two modes that needs to be clarified. The key difference is that 360-degree VR presents *real-world video footage* of a particular environment that has been pre-recorded, whereas animated VR presents *animated* (i.e. images that are computer generated) scenes of environments that can change in real time according to how the user interacts with virtual objects in the environment [12]. Animated VR allows for users to interact with virtual objects and to influence the course of events in real time. However, 360-degree VR presents video footage that is pre-recorded and fixed in its current state, and thus users can only watch the footage and cannot use physical actions to influence the situation being presented in any way.

### Animated VR

At one end of the XR spectrum lies animated VR, which can be defined as a computer-simulated environment that aims to simulate a sense of being physically and psychologically present in another place by completely occluding the real world [13, 14]. In animated VR, the user experiences complete immersion in the animated virtual world whilst being completely blind to the real-world environment [15]. This experience occurs within a head-mounted display (e.g. HTC Vive headset) wherein the user receives sensory input (visual and auditory) from the headset display and connected speakers/headphones, rather than from their real-world surroundings. However, users still pick-up haptic feedback and some contextual information from the real world (e.g. the type of surface they are standing on whilst in the animated VR environment, and air temperature). Animated VR can also be presented on large screen displays (flat or curved screens), which require users to wear three-dimensional glasses, as well as on Cave Automatic Virtual Environment systems, otherwise known as CAVE. Animated VR has the advantage of allowing users to physically interact with features of the animated environment (virtual objects) [16, 17] and to directly influence the course of events as they unfold. In addition, any form of contextual information within the virtual setting (e.g. appearance and behaviour of objects, environment conditions, tactics used by virtual opponents) can be manipulated. This provides freedom to manipulate any constraint in the environment and allows specific situations to be created. However, a limitation of this technology is that it can be difficult to simulate virtual objects that behave the same way as in the real world, and thus the nature of the perception–action couplings performed by users may not be representative of the real performance environment [16, 18].

### 360-Degree VR

Sitting alongside animated VR on the XR spectrum is 360-degree VR, which presents real-world video footage of a particular environment that has been pre-recorded using a 360-degree video camera and is fixed in its current state (see [12] for an example in Australian Rules Football umpiring). 360-degree VR is positioned alongside animated VR on the XR spectrum because it presents 360-degree video using a VR headset (e.g. Oculus Quest) which completely occludes the real world in the same way as animated VR. Advantages of 360-degree VR include its capability of completely immersing users into the situation being viewed, users can perform head movements to view the 360-degree scene, and the perceptual information presented is often highly representative because it typically involves visual information that has been recorded or sampled from the real-world setting. However, key limitations are that the tool does not allow the user to influence the situation being presented in any way, thereby creating a decoupling of perception and action that may be detrimental to skill development [9].

### AR

While animated VR and 360-degree VR completely occlude the real world via the use of headsets (with the exception of CAVE systems), further along the spectrum is AR technology whereby virtual objects can be overlayed in the real-world environment. Augmented reality is an experience of a real-world environment where objects in the real environment are enhanced or added by computer-generated perceptual information including visual and auditory sensory modalities [19, 20]. The virtual sensory information overlaid in the real world can be additive in nature (i.e. stimuli that are added to the task being performed in the natural environment) or it can be masking (i.e. occluding/hiding certain elements of the natural environment), which is achieved either through a hand-held device (e.g. smart phone or tablet) or Smart Glasses that can be water resistant (e.g. SOLOS Smart Glasses, Form Smart Swimming Goggles) [these devices are a similar size and shape to everyday reading glasses or swimming goggles]. These devices present images of virtual objects on top of the real-world environment in the users’ field of view (e.g. a swimmer’s pace per 100 m can be presented in their field of view in real time whilst swimming). A notable disadvantage of AR is that it does not allow users to physically interact with or change the position or appearance of virtual objects that appear in the natural environment (if it does allow this it is closer to MR), and the realism of objects (e.g. brightness) can be low. Moreover, a disadvantage of AR is that the virtual objects and real-world environment do not interact with one another (e.g. a virtual ball will not change its velocity if it is seen to collide with a real wall, but instead, it will go straight through the wall because it does not recognise that the wall is there).

There have been various AR applications developed that can be used with smart phones and tablet devices. The most popular applications in recent times have included Pokémon Go, which allows people to use a built-in camera in a device such as a smart phone to view virtual objects as if they were actually present in the real world. Other AR programmes are capable of overlaying life-sized virtual objects in the real-world setting through smart glasses (e.g. a virtual player performing a set in volleyball); however, they do not allow the user to interact with the visual information (e.g. virtual volleyball).

### MR

At the opposite end of the XR spectrum is MR, which places virtual objects over the real-world environment in a similar way to AR, but with the addition of users being able to physically interact with these virtual objects. This creates a new reality whereby real and digital objects co-exist and interact with each other in real time. Users experience MR using headsets such as the Hololens, which allows virtual objects to be viewed over the top of real-world objects, enabling users to interact with the objects using their hands or feet. For example, a car mechanic can be looking at a virtual life-sized car using the Hololens headset and be able to use their hands to physically spin the car around or lift the hood of the car to view and manipulate the inside parts (e.g. Microsoft Mesh). Similarly, the company ‘Saab’ has created an MR tactical display where digital landscapes of specific environments and objects can be viewed and manipulated (e.g. the tactical positioning of submarines in the ocean or the position of soldiers on the ground). Other potential applications include a virtual combat environment that can be placed in the real-world environment, such as virtual people attempting to attack the users with weaponry. A potential application in sport could be volleyball players practicing their serves or spikes whilst viewing virtual opponents on the other side of the net who can intercept the ball (i.e. virtual blockers). Importantly, all virtual objects in MR interact with the real-world objects in a seamlessly natural way. This allows for contextual information in the environment to be manipulated whilst maintaining a higher degree of representativeness (compared with other forms of XR) with how virtual objects behave in the real world. A limitation of MR is that virtual objects can be slightly delayed in the way they move when users interact with them, which can decrease the representativeness of perception–action couplings performed by users, and haptic feedback is unlike the real world (i.e. the use of vibrations). Additionally, the realism and brightness of virtual objects when using MR can be low.

## Theories Underpinning the Development of XR Tools

The following section aims to explore theoretical principles and approaches that can aid the design and assessment of XR tools. A key issue preventing high-performance sporting organisations from investing the time and resources required to develop and implement XR training tools is the lack of understanding about the capability of XR to train perceptual-motor skills and elicit positive skill transfer. One way of improving this understanding is to draw upon the theoretical framework of ecological dynamics to underpin the application of skill acquisition principles [7]. This theory can help inform sports about the key factors to consider when designing, adopting or assessing XR tools in order to optimise skill transfer.

### Ecological Dynamics

The theory of ecological dynamics is derived from ecological psychology and dynamics systems theory and describes the mutuality of the performer-environment relationship [9, 21]. Through an ecological dynamics lens, athletes and sports environments are viewed as complex adaptive systems, where athlete behaviour is shaped by the continuous interactions with task, environmental and performer constraints that exist within the performance environment [22]. As performers adapt to their environment, they become attuned to what actions are supported by the available information [7]. Over time, learners attend to more useful information, which is more reliable for action selection and control [21].

The control of goal-oriented action is dependent upon the process of individual self-organisation and the constraints imposed upon performers [23]. Constraints can be classified as individual (e.g. physical and psychological characteristics), task (e.g. rules and equipment) and environmental (e.g. socio-cultural, weather, light) [24], and are described as the boundaries within which human neuromusculoskeletal systems operate, therefore shaping the emergence of patterns of coordination and control [23]. Constraints can also be dynamic and emerge and decay with time, for example, fatigue, the time left on the game clock, and emotions that can affect perception and action [7]. Therefore, optimal movement solutions emerge from the interaction of constraints with self-organising processes [25, 26].

Consequently, effective training designs need to include key task, environmental and individual constraints to allow athletes to discover functional adaptive movement solutions that can be successfully applied in the performance setting [7]. A key challenge for XR programmers and practitioners is to sample the critical sources of information from the competition setting and include these in XR practice tasks to ensure constraints are representative of the performance environment [9, 27, 28]. Successfully sampling this information increases the representativeness of training tasks by maintaining perception–action couplings, thereby helping athletes to attune to relevant information to generate movement solutions [7, 29].

### RLD

Representative learning design was adapted from Bunswik’s experimental design framework to help guide the design of practice tasks that promote skill transfer to the competition setting [9, 27]. Representative learning design highlights the importance of considering interacting constraints on movement behaviours and coupling perception and action processes during practice tasks [7, 11, 30, 31]. Sampling key constraints from the competition setting is a way to faithfully maintain key perception and action behaviours, and in turn, promote transfer [9, 11, 27].

To guide practitioners and researchers in the development and assessment of training tasks, RLD argues that the concepts of functionality and action fidelity are essential for optimising transfer [9]. Functionality refers to athletes basing their decision making and actions on comparable information to that of the real competition environment [9]. Comparatively, action fidelity refers to whether a performer’s action or behaviour remains the same in the training and performance environment [32, 33]. In this sense, RLD promotes practice tasks with sport-specific action responses rather than verbal responses or button/joystick pressing [9, 10]. Therefore, to enhance the representative design of XR training, practice tasks should sample the information and action responses that are found in specific situations within the performance environment to ensure the functional coupling of key perception and action processes [9]. Doing so would allow performers to regulate their movement behaviours in practice based upon comparable information found in the performance setting, thus helping to ensure that the learning that takes place in the XR environment is based upon athletes coupling their actions to relevant sources of information [9, 32].

This section introduced ecological dynamics and the underpinning principles that can help guide the design and assessment of learning tasks that promote skill transfer. The next section explores how these theoretical principles can be operationalised to inform the assessment and design of perceptual-motor training tools such as XR.

## Methods Used to Assess the Efficacy of XR Tools

### MPTF

Underpinned by key concepts of RLD, the MPTF [10] offers a method to assess the effectiveness and to provide a guide in the design of perceptual-motor training tools, such as XR. The MPTF aims to provide learning designers with a tool to predict the degree to which a modified perceptual training approach will transfer to improvements in on-field performance [10]. The tool considers three key factors including the perceptual skill to be trained, the stimulus correspondence and the action correspondence [10]. Correspondence is a reference to the functionality of the stimulus (type of information presented) and the representativeness of actions performed during the task and the potential for transfer [9, 10]. Stimulus correspondence can range from generic (e.g. arrows indicating the direction of player movement) through to sport specific (e.g. opponents), while action response can range from generic (e.g. verbal or button press responses) to sport specific [10]. The MPTF posits that greater transfer is likely to occur when the stimuli and actions performed are closer to the sport-specific end of the continuum [10]. Practically, this suggests that XR training tasks should (1) aim to sample relevant sports specific information, and (2) require performers to respond to this information as they would in the normal competition setting (10, 9].

### Fidelity, Construct Validity and Transfer Assessments

Although the terms action fidelity and functionality have been described in the RLD section above, these terms (or the main idea of these terms), have also been described by researchers within the XR domain [34, 35]. For example, XR researchers have created sub-categories of fidelity (e.g. physical, psychological, emotional and action fidelity), which are deemed to be important for eliciting skill transfer [34, 35]. However, it is important to note that these sub-categories describe similar concepts seen within RLD (i.e. functionality and action fidelity) and portray the same key message of maintaining representativeness, that is, understanding the degree to which XR simulations faithfully simulate information and action [9]. For this reason (and to avoid confusion), we will not describe the sub-categories of fidelity detailed in the XR domain, but readers can refer to [34, 35] for detailed explanations. We now define the terms fidelity and construct validity as they have been applied in XR environments.

The terms fidelity and construct validity are terms used within the XR domain that describe the qualities of an XR tool and the extent to which that tool is likely to promote positive transfer to the target setting [34]. When used in reference to XR tools, fidelity is defined as the extent to which an XR tool represents the real-world environment, in terms of the appearance, cognitions, behaviours and affective states it elicits [34, 36], and is therefore closely linked with the idea of RLD [9]. The term construct validity relates to the degree to which the XR tool is able to distinguish between users of differing expertise levels [37]. Expert-novice discrimination has previously been used to validate VR surgical simulations [38, 39], and is considered to be an indicator of whether important features of the real-world task have been captured within the simulated version of that task [35]. It is assumed that if the key performance indicators used when performing a task in XR are valid and reliable measures of real-world ability, then that tool should be able to reliably distinguish between expert and novice performers [38, 40]. Therefore, if training tasks developed within the XR environment truly represent the skills required in the real-world setting, then athletes that excel in the real world should also excel when performing in XR [34]. Thus, the assessment of construct validity is considered by XR researchers to be an important step in evaluating the representativeness of an XR training tool [35]. Notably, however, just because experts perform better on an XR task compared with their lesser skilled counterparts, does not automatically mean that training using the XR tool will result in positive skill transfer to the real-world setting. Rather, this simply indicates that there is likely to be some degree of overlap between the perceptual-cognitive and motor skills needed to perform well in both environments (i.e. XR and real-world equivalent environment) [41].

Ultimately, the efficacy of XR tools will be determined by the degree of skill transfer that is seen from the XR training setting to the competition setting. Transfer tests are the final method that can be used to assess the utility of an XR tool and predict the effectiveness of XR training. Extended reality learning tasks can be designed with the aim of enhancing a particular skill that is deemed important for performing well during competition (e.g. passing accuracy) [42]. Athletes’ performances are measured before and after the training intervention and compared with a control group to assess the effect of the training on performance during competition. The real competition setting is the ideal place to assess skill transfer [6] as the aim of any training intervention is to ultimately enhance performance in this setting. However, some researchers have designed small-sided games that provide specific situations for particular skills to be measured more easily and frequently compared to the competition setting [43, 44]. Sport-specific examples of XR training interventions that measure skill transfer are provided during the XR training interventions section of this review.

In summary, the MPTF and assessments of fidelity and construct validity can provide information to make informed judgements about the probability of transfer from an XR task to a performance setting. Transfer tests will ultimately provide the final judgement about the effectiveness of the XR tool for enhancing performance during competition. The next section explores the use of XR tools in sport, including how they have been assessed for their level of representativeness (the extent to which XR represents the real-world setting), and how they have been adopted for use in training interventions.

## Efficacy of XR Use in Sport

Although there have been many XR tools developed to train skills in sport, the majority of tools in the broader sports community have not been assessed for their level of representativeness. This is a significant issue because the use of XR tools in the practice setting that do not adequately represent the real-world task may result in negative skill transfer and/or a decrease in performance in the real-world setting (e.g. changes to movement timing in interceptive sports), as well as wasting valuable time and resources. Therefore, it is vital that XR tools are assessed for their level of representativeness (before they are used during training) using measures including construct validity and transfer tests. Additionally, the skill acquisition principles and frameworks discussed earlier provide further guidance for how XR tools can be developed and assessed. Collectively, these assessments will help to determine the likelihood of the XR tool eliciting positive skill transfer to the real-world performance setting, and can help determine the changes that need to occur within the design of the XR tool to increase its level of representativness before it is used for training. We now provide examples of how XR tools have been assessed in the past and highlight theoretical limitations associated with these assessments.

### Assessing the Representativeness and Construct Validity of XR Tools

Although the majority of XR tools have not been subjected to a formal assessment, there are some exceptions within the sporting domain in sports including, golf [45], tennis [46], baseball [47], soccer [41], volleyball [48] and rugby [49]. We have grouped this research into two main themes including (1) construct validity assessments and (2) representativeness assessments.

#### Construct Validity Assessments

All of the construct validity assessments within the XR literature have been conducted using animated VR simulations where participants (experts compared to lesser skilled performers) have performed tasks such as golf putting [45], soccer goalkeeping [50], small-sided games in soccer [41] and defensive interception in rugby [49]. The tasks used to assess construct validity have ranged from interceptive and aiming tasks that are relatively static in nature (e.g. golf putting) [45] to dynamic tasks that require greater movement within the VR environment (e.g. performing dynamic ball movement drills in soccer) [41]. Most of these tasks have coupled perception and action [41, 45, 49, 50], with one study using a perception only task [49] (study one). The measures that have been used to assess differences in expertise have included putting accuracy [45], response time when intercepting (catching) an oncoming ball in soccer [50], a combination of passing accuracy, composure, reaction time, and adaptability when performing soccer drills (a diagnostic score relating to the predicted expertise level of each player was calculated by combining these measures) [41] and anticipation accuracy when defending in rugby [49]. Some of these tasks have included real-world kinematic information that was captured in the real-world setting and imported into the VR system to enhance the representativeness of the simulation (e.g. the real-world running kinematics of rugby players [49], and some tasks have artificially created perceptual information in an attempt to re-create real-world contextual information (e.g. soccer ball trajectories) [50].

The findings have revealed differences between experts and novices across several motor tasks [41, 45, 50]. For example, expert golfers have displayed superior putting accuracy compared with novices [45], elite goalkeepers outperformed novices by waiting significantly longer before initiating their movement to catch a ball [50] and expert soccer players have outperformed novices on three out of four soccer game-based drills (the one drill where expertise differences were not found was later identified as not being representative of real-world soccer) [41]. Moreover, a perception-only task in rugby found that experts could accurately detect, early in the movement, the final running direction of the attacking player significantly more accurately than novices [49]. Additionally, experts were able to determine the non-deceptive body movements of attacking players to facilitate anticipation, compared with novices who focussed upon the deceptive signals of the attacking players [49]. A follow-up study that coupled perception and action revealed that experts made fewer movement errors in the wrong direction and had a significantly smaller final distance between them and the virtual attacker [49] (study two). Collectively, these results are promising for the capability of animated VR to differentiate between experts and novices, which may suggest that the information sources used by experts to enhance their performance in the real world are also evident within the animated VR environment. However, it may be that experts in these studies were simply better at adapting compared with novices, and therefore could identify and utilise information to facilitate their actions and decision making to enhance task performance (i.e. general transfer effects). Similarly, and as highlighted earlier, the existence of expert-novice differences does not necessarily indicate that the training tool will result in positive transfer of the trained skill to the real-world performance environment. Further research is therefore needed to improve the measures used within construct validity assessments to better understand the reasons *why* expertise differences are found within XR environments.

#### Representativeness Assessments

Extended reality tools have been assessed for their level of representativeness in sports including, tennis [46] and baseball [47] using animated VR, with one study in volleyball using AR [48]. Notably, all of these tasks allowed users to interact with features of the virtual environment (i.e. virtual tennis balls) by performing an action (i.e. moving into position to perform a forehand groundstroke), thereby maintaining perception–action couplings that were more likely to be representative of the target setting. The measures that have been used to compare the representativeness of the XR tool and real-world setting have included assessing the number of steps and type of stance used when performing groundstrokes in tennis (players reacted to the same ball trajectories in both real-world and animated VR conditions via the use of Hawkeye technology) [46] comparing swing velocity when swinging at curved and fast balls in an animated VR baseball environment and real-world baseball environment [47], and comparing patellar tendon loading and landing force when performing a spike action (including movements in the lead up to the spike) in volleyball [48]. The results of each of these studies found no significant difference between the actions performed in the XR environments and real-world settings for all sports tasks, suggesting the actions performed in XR were highly representative. Collectively, these results imply that performers were able to judge the trajectory of the balls in tennis [46], baseball [47] and volleyball [48] in a similar manner in both XR and real-world conditions, suggesting the information performers were using to inform their actions was highly representative. The volleyball study specifically provides evidence that AR can replicate biomechanical outcomes designed to be more representative of the real-world task compared with traditional laboratory-based testing settings, with the additional advantage of creating more sport-specific scenarios without the need for other players to be involved [48]. Other sports may be able to utilise AR in the future by providing sport-specific contextual information within laboratory-based settings to assess biomechanical behaviours. Although these collective findings are promising, further research is needed to establish more rigorous ways of assessing the representativeness of actions performed when using XR tools, such as biomechanical analysis of actions using motion capture technology (e.g. Vicon). In addition, more comprehensive assessments are needed to ensure that XR training environments do not elicit negative skill transfer, that is, any unwanted changes to perception–action couplings that could decrease performance.

It must be noted that the studies described above have their limitations and shortcomings, particularly when examining them through the lens of RLD. Many of these studies have only addressed one aspect of the XR tool when assessing its representativeness. To truly understand the potential influence of a given XR tool on performance, tools need to undertake a more complete assessment that investigates the functionality of perceptual information and actions performed, and how these two elements are coupled by athletes to perform functional goal-directed movements that can be adapted to various situations. Comparing how athletes adapt their behaviours in XR vs real-world conditions when performing under the same task constraints in both environments would be an interesting area for future research. Moreover, understanding the degree of representativeness required for positive transfer may also be important because some XR tools may afford greater transfer than others, depending upon the extent to which they faithfully simulate the information present in the performance setting. Finally, research has yet to examine how differences in the affective demands (e.g. stress/pressure, arousal level) of tasks within XR and real-world environments influence how athletes adapt their behaviour.

### XR Training Interventions

Although there has been much interest in the use of XR technology for training skills in sport, there have been few studies published on this topic to date. From the small number of training studies that have been published, the majority have utilised either 360-degree VR or animated VR to train athletes, with fewer studies utilising AR.

#### Perceptual-Cognitive Skill

The perceptual-cognitive skill of athletes has been trained by presenting 360-degree video of real game footage using a head-mounted display [42–44]. Pagé et al. [43] compared the effectiveness of 360-degree VR and two-dimensional (2D) video against a control group to train the decision-making skills of basketball players when performing specific plays. Decision making was assessed on-court before and after training sessions using two types of plays including trained plays (presented during training sessions) and untrained plays (presented during the on-court tests only). Results showed that the VR training group and a 2D video training group significantly outperformed the control group when facing the trained plays in the post-test. When facing the untrained plays, the VR training group outperformed the 2D and control groups. These results suggest that 360-degree VR training may provide a better means of improving decision making for real-world situations compared with 2D video training. The next step would be to assess the relative effectiveness of 360-degree VR training over typical real-world decision-making training that is done in the real-world training environment.

Fortes et al. [42] compared the effectiveness of 360-degree VR and 2D video for training the decision-making skill of national level junior soccer players. Players underwent 18 training sessions over a 6-week period. Results showed that both training groups significantly improved passing decision making (defined as passes that went to a free teammate, directly or indirectly created a shot attempt, or went to a player in a better position) during real-world small-sided games. Notably, the 360-degree VR group improved decision making significantly more than the 2D video group. This result suggests that greater immersion in the training stimuli via the VR headset (which allowed participants to swivel their head), may have led to a greater improvement in passing decision-making compared with 2D video. Interestingly, skill transfer occurred despite players not coupling perceptual information with a representative action during training. Future research is needed to establish why this occurred, and to also compare the representativeness of coupled vs uncoupled actions during training to examine whether prolonged use could lead to negative transfer effects. In addition, and in a similar vein to the research reported by Pagé et al., [43], while the 360-degree VR training showed superior results compared with 2D video-based training, it is important to also compare VR training with real-world training to determine the extent of the similarities between the two approaches.

Panchuk et al. [44] trained the decision-making skill of highly skilled male and female basketball players by comparing the effectiveness of 360-degree VR training against a control group who participated in their usual on-court training routine. The training group completed an average of 11 sessions across 3 weeks in which they viewed gameplay scenarios. Players in the training group were asked to verbalise their decision making as quickly as possible while also using a basketball to mimic the decision-making action (although they did not actually release the ball from their hands). The results showed that male players in the training group had a medium to large, but not statistically significant improvement in performance (*p* = 0.080, *d* = 0.74) during an on-court small-sided game transfer test compared with the male control group. Interestingly, the female group improved significantly at the VR training task; however, this did not transfer to the small-sided games transfer test. The non-improvement of the female training group may have been due to one of the limitations of the experiment, the use of only male players in the training stimuli. This meant the female training group practiced attuning to male players during the training intervention, but were assessed for transfer in small-sided games against female individuals. However, the results may have been equally influenced by other variables such as participants’ playing experience, skill level or anthropometrics. Additionally, 360-degree VR training was not detrimental to real-world performance during small-sided games. Therefore, given the affordability of implementing this type of training, Panchuk et al. [44] suggested that 360-degree VR could be beneficial for keeping players cognitively engaged and at least maintaining decision-making performance when injured or whilst travelling [44]. However, given that the effect of prolonged use of such tools is unknown (i.e. prolonged use may result in negative skill adaptations), further research should explore the long-term effects of training using XR tools such as 360-degree VR.

The use of animated VR for training perceptual-cognitive skill is extremely rare in the literature. In one of the few studies to employ this method, Gray [6] used an animated VR tool to investigate transfer of training from a VR baseball simulator to real-world baseball performance. Participants were assigned to four groups including adaptive hitting training using VR (where the pitch type was systematically varied to consistently challenge the batter), extra sessions of batting practice in VR, extra sessions of real batting practice using a pitching machine, or a control condition that required participants to simply complete their usual training sessions (20 participants were assigned to each group). Training involved two 45-min sessions per week for 6 weeks. Results showed that the VR adaptive training group significantly improved from pre-test to post-test on a VR batting test, on-field batting test and a pitch recognition test. Additionally, the VR adaptive training group showed superior batting statistics in the competitive baseball season after the intervention and reached higher levels of competition across a 5-year period. These results suggest that VR animated training can be used to improve real-world performance, particularly when contextual information is manipulated in the animated VR environment to provide a more variable practice task (i.e. adapting pitch type) [6]. However, given that the real batting practice group in this study used a pitching machine, it remains to be seen whether the adapted VR training would provide superior performance outcomes when compared with a period of batting practice against actual pitchers who also varied their pitching.

Animated VR was used by Tsai et al. [51] to train basketball players to learn tactics and implement those tactics correctly during real-world situations. The study compared the training effectiveness of more conventional tools used to teach tactics in basketball (e.g. white board and 2D video screens) with an animated VR simulation. Each learning group completed one training session (20 min in duration) where they learnt four different basketball tactics from a first person’s point of view. The VR group showed a significant improvement from pre-test to post-test (which was completed on a real-basketball court) in terms of their movement pattern (correctness of running position) compared with the other groups who showed no change in performance. However, this result only occurred on one of the more complex tactics, which consisted of more players and more difficult running paths and thus the authors concluded that VR tactical training was effective for learning more complex tactics. A limitation of this study was that participants performed movements in the VR environment using a remote-control device, which therefore reduced the representativeness of the actions performed in the task. Nevertheless, this study provides a foundation for future work to explore the effectiveness of animated VR for training tactical decision making.

In summary, XR appears to have the potential to be a promising tool for training perceptual-cognitive skills in sport; however, improvements in research design (e.g. inclusion of adequate transfer tests, control conditions and comparisons to representative practice tasks) are required to provide more clarity on its effectiveness over and above normal physical practice. Further research is warranted to establish the representativeness of athletes’ perception–action couplings in the XR environment and how this effects skill transfer, as well as further exploring the advantages that XR may provide in terms of manipulating contextual information. Investigating whether any negative skill transfer occurs from the long-term use of XR tools in training is critically important.

#### Motor Skill

The use of VR technology to train motor skills has been growing in popularity over the past decade. Juggling has been a prevalent motor skill that has been used to study the effects of animated VR training on real-world performance [52, 53]. Borglund et al. [53] trained two groups of participants to juggle with two balls using an animated VR simulation. Participants in the feedback training group received timing and height performance feedback throughout the training session (200 trials and approximately 30 min in duration), whilst the control group performed the same task with no timing and height performance feedback. Performance feedback was presented using two bars with a green and red zone. Two crosses represented the height of the throws from the right and left hand, respectively. For example, if the cross appeared below the midline in the green zone, this meant the altitude was too low. Similarly, timing feedback was presented with a cross on a horizontal bar with green and red zones representing the timing of throws performed with each hand. The results showed a significant decrease in the number of dropped balls from the start until the end of training for all participants. Additionally, all participants improved significantly on measures including juggling height and timing across the training session. However, there were no statistically significant differences found between the feedback and control groups, indicating that the time and height feedback did not elicit additional performance improvements, compared with no feedback. These findings highlight the potential of animated VR technologies to improve motor skill performance in the future.

Skill transfer from virtual to real-world environments has been examined in darts by measuring the throwing accuracy and quiet eye duration of novice performers [54]. Participants in the VR training group and real-world training group completed three training sessions that consisted of performing 50 throws towards a virtual or real dart board. The VR training group exhibited a significantly longer quiet eye duration compared with the real-world dart throwing group, but as highlighted by the authors, a key limitation of this study was the lack of similarities between the motor requirements for the real and virtual conditions [54]. In addition, the authors further highlighted differences in the aiming requirements for both tasks [54]. The VR group were able to use a crosshair to assist with their aiming, which may have resulted in the increased quiet eye duration for this group [54].

Rauter et al. [55] investigated the level of skill transfer from training using a virtual rowing simulation. Recreational rowers were immersed in a CAVE display where they were surrounded by three 4 m × 3 m screen projectors that displayed visuals of the water. Additionally, the simulation included sounds of the boat moving through the water, and the use of oars that were attached to ropes which produced haptic feedback. Results revealed that the VR training group significantly improved from pre-test to post-test on several biomechanical measures of technique when tested on real water. Additionally, this study included a real-water training group that showed significant improvements in biomechanical technique when tested in the VR CAVE environment, providing evidence for a transfer in both the real and virtual environments. This pilot study (four participants per group) provides interesting results, but is limited by a small sample size and lack of a measure of a far transfer (e.g. time to complete a rowing race) [6].

Research findings have suggested that performers can learn simple motor tasks in VR and real-world training settings at a comparable rate [56, 57]. However, it is worth noting that rapid improvements in performance do not necessarily mean that other benefits also occur such as retention and transfer. Moreover, while skills learnt in VR have been found to improve performance when switching to a real-world setting, the performance following training in VR is poorer compared with the performance when someone has trained in a real-world setting from the outset [57]. Additionally, studies suggest that improvements in skill that are made within the VR environment do not always generalise to real-world settings [58]. In fact, a recent study demonstrated that playing a generic dart game available on an HTC Vive headset resulted in a worsening of performance compared with real-world training [58]. This decrease in performance likely occurred because the action of throwing a dart in this VR game was not representative of a real-world dart throw. Therefore, this highlights the importance of evaluating the extent to which an XR tool is able to represent the perception–action couplings that exist in the real-world sport setting.

Overall, the efficacy of improving motor skills through training in VR has been low. More research is necessary to study motor learning in different XR systems, as well as to establish how learning using these systems translates to real-world performance [59]. A key question is to what extent motor skills can be simulated in the XR environment with high representativeness. It is possible that motor skills that rely more on ‘feel’ or precise haptic feedback for effective execution (e.g. performing a set in volleyball) are unable to be replicated in the XR environment with high representativeness, whilst other motor skills that require less precise haptic feedback for effective execution can be simulated more successfully (e.g. a block in volleyball).

Further research is clearly needed to compare different types of motor skills under different situations in the XR and real-world environments. We now shift our focus to the future utilisation of XR by outlining how research from other domains can inform coaches and practitioners, providing future research directions and practical applications, and explaining some areas for consideration relating to the use of XR for training skills in sport.

## Future Utilisation of XR in Sport

### Learnings From Other Domains

Extended reality technology (particularly animated VR) has been used for decades within other domains including aviation [60], medicine [61] and the military (see [62] for a review). This research can provide a foundation from which the sports domain can help to facilitate the application of XR into the future. For example, XR has been used to train the skills of first responders including police officers, paramedics and firefighters [63]. Harris et al. [63] examined the effectiveness of animated VR for training room searching procedures for police officers and assessed the corresponding development of perceptual-cognitive skills through eye-tracking indices of search efficiency. Fifty-four participants were assigned to a VR rule-learning and search training task, a search only training task or a no-practice control group. Both the VR and search only groups developed more efficient search behaviours during the animated VR training task. However, although more efficient gaze behaviours were performed during training, these were not evident during the transfer test. These results highlight the challenges of achieving a skill transfer from animated VR training to the real world, which is also a current challenge in the sports domain.

Koutitas et al. [64] used VR and AR to train the crews of an ambulance bus (a large ambulance equipped with more sophisticated medical equipment) to enhance their level of expertise. Participants were divided into AR, VR and PowerPoint presentation training groups. The results showed that after 1 week of training, the error rate in the AR and VR groups was significantly less than that in the PowerPoint presentation group. This result highlights how XR technology may be a better alternative compared with more traditional training methods. Indeed, a similar method within the sport domain is the use of whiteboards or PowerPoint presentations to describe team tactics, which could be replaced by XR technology to create a more immersive and hands-on learning experience [51]. Interpreted through the MPTF, these findings might be a result of the information stimulus in the XR conditions having higher representativeness.

Virtual reality has also been used for treatment, therapy and surgery in the medical field [65, 66]. Previous work has suggested that using a well-designed representative training simulation can significantly reduce errors of healthcare workers and improve patient safety [65]. Notably, because of the importance of tactile feedback in medical professions, most training tools require tailor-made equipment. For example, Gallagher et al. [67] and Grantcharov et al. [66] used a VR device outfitted with two laparoscopic instruments that the surgeon manipulated to simulate a surgical procedure. In this subfield of training, researchers must not only provide a highly representative visual experience, but they must also develop realistic and responsive physical devices to enable realistic actions. As per the MPTF [10], if XR tasks afford more representative actions, this is likely to increase the probability of transfer. These studies provide examples of how sport-specific XR equipment can be tailored to the needs of sports. Indeed, a recent study from Le Noury et al. [46] created a tailored tracking device that could be placed on a real tennis racquet using a three-dimensional printer, which enabled tennis players to use their own racquets to hit virtual tennis balls.

Animated VR has also been used as a tool during exposure therapy treatment for conditions such as phobias [68–70]. A strength of animated VR in this case is that it provides an environment that contains the phobic stimulus; however, the environment is safe for the patient. Virtual environments can be created to provide exposure for situations that are more challenging to create in the real world, such as fear of flying [71], agoraphobia (anxiety disorder whereby people avoid certain places or situations) [72] and height phobia [73]. Findings have demonstrated positive effects from this type of treatment across a range of different phobias [74]. Sport-specific animated VR environments could be used to expose athletes to conditions that induce anxiety or stress similar to that experienced in the competition setting (e.g. a volatile crowd environment), which in conjunction with a psychologist, may help athletes learn strategies to overcome such challenges. Furthermore, athletes who have negative experiences when competing in sport (e.g. a crash on a Bobsled at the Winter Olympics that causes a serious injury) may be able to use animated VR as a treatment tool to help them recover psychologically and return to the sport sooner.

The military have used animated VR for simulating stressful situations and managing responses through stress management training [3, 5, 75]. A review of VR-based stress management training programmes conducted by Pallavicini et al. [76] concluded that animated VR can help military personnel cope with emotional and physiological responses to stressors in order to maintain performance in stressful situations [77]. Interestingly, animated VR appears to be a promising tool to assess individuals’ resilience to stress and to identify the impact that stress can have on physiological reactivity and performance [78]. Stressful scenarios simulated through VR technology can also be used to assess physiological responses to stressors and connect these specific responses to task performance [79]. This allows the military to identify resilient individuals or those at risk of stress-related performance issues and offer supplementary training as necessary. High-performance sport organisations may be able to use animated VR in a similar way by identifying athletes at risk of experiencing stress-related performance issues, and therefore provide additional training where necessary.

Animated VR can also provide interactive stress management training that is useful for decreasing levels of perceived stress and negative emotions in military personnel, as well as treating post-traumatic stress disorder (PTSD) [3, 75, 79, 80]. Results have shown that after a 5-week period of VR exposure therapy treatment (11 sessions of treatment on average), 16 out of the 20 soldiers no longer showed significant signs of PTSD, and a significant reduction in depression and anxiety was evident [3, 81]. These improvements were also retained after a 3-month follow-up [3, 81]. Interestingly, these participants had been previously treated for PTSD using other commonly used methods but had experienced no significant benefit. Additionally, animated VR environments coupled with arousal reduction strategies (e.g. relaxation techniques, biofeedback techniques and desensitisation through exposure to stressful situations) have been found to effectively increase resilience to stress [3, 76]. Based upon these findings, exposure therapy using animated VR has the potential to be utilised in sport by giving athletes exposure to challenging situations they may face in the future to help ease anxiety [3, 76, 82].

The evidence showing the effectiveness of animated VR for treating PTSD and phobias is supported by the principles of affective learning design, which argues that emotions are an integral part of learning in sport [28]. Practicing in environments that simulate the affective demands of competition is considered important to facilitate skill transfer, particularly to high-pressure environments [83]. For example, practicing sporting skills under anxiety improves the stability of these skills in high-anxiety-inducing situations, such as competition [83, 84].

### Practical Applications

Current research findings suggest that XR has the potential for training perceptual-cognitive skills in sport. It is clear that the effectiveness of XR training tools is largely dependent upon the level of representativeness of the tool, suggesting that this should be assessed before the training tool is used within a skill development programme. At present, it may be better to integrate XR training sessions within a regular on-court or on-field training programme with the aim of adding additional value to regular training. However, given the low level of evidence currently available, XR training should never replace training in the real-world environment for healthy athletes. An exception to this rule may apply to athletes who are not able to participate in regular training activities because of an injury. These athletes may use XR tools as part of their rehabilitation programme (assuming the tool is highly representative of the target setting) with the aim of enhancing, or at the very least, maintaining perceptual-cognitive skills (e.g. tactical decision making, anticipation). However, further research is required to determine whether prolonged use (or even short-term use) of this form of XR training leads to a negative transfer, particularly if athletes are not completing any real-world skill training when using XR.

A fruitful area where XR can be utilised in sport is to help develop tactical skills or educate athletes about tactical decision making [51]. Developing XR tools that evolve the current methods used to educate and train athletes’ tactical decision making (i.e. the use of Power Point, reviewing video clips of past performance) is an area that has enormous potential. Just as first responder training was shown to be more efficient with XR training vs a Power Point presentation [64], tactical education sessions could be undertaken using VR technology. Multiple players (20 or more) and a coach can wear a VR headset and be transported to the same virtual space at the same time (e.g. a virtual basketball stadium). Inside the VR environment, players can view an opposition team’s defensive zone structure (via pre-recorded GPS data from past performances) from a player’s on-field viewing perspective. The coach is able to simultaneously transport all players to various positions in the playing area and give players a live view of how, for example, the structure of the defence changes depending upon the location of the ball. This allows players to learn tactical information in a more immersive and interactive setting compared with current methods.

Furthermore, given previous research has shown that animated VR can be utilised in fields such as psychology and the military to simulate stressful situations [79, 80], these training approaches could be applied in a similar manner to improve the performance of athletes in situations of heightened emotions. Sport-specific situations that produce high stress for athletes could be simulated to give athletes greater exposure to these environments with the aim of helping to limit performance decrements under high-stress conditions in the real competition environment [76, 82].

**Fig. 2:**
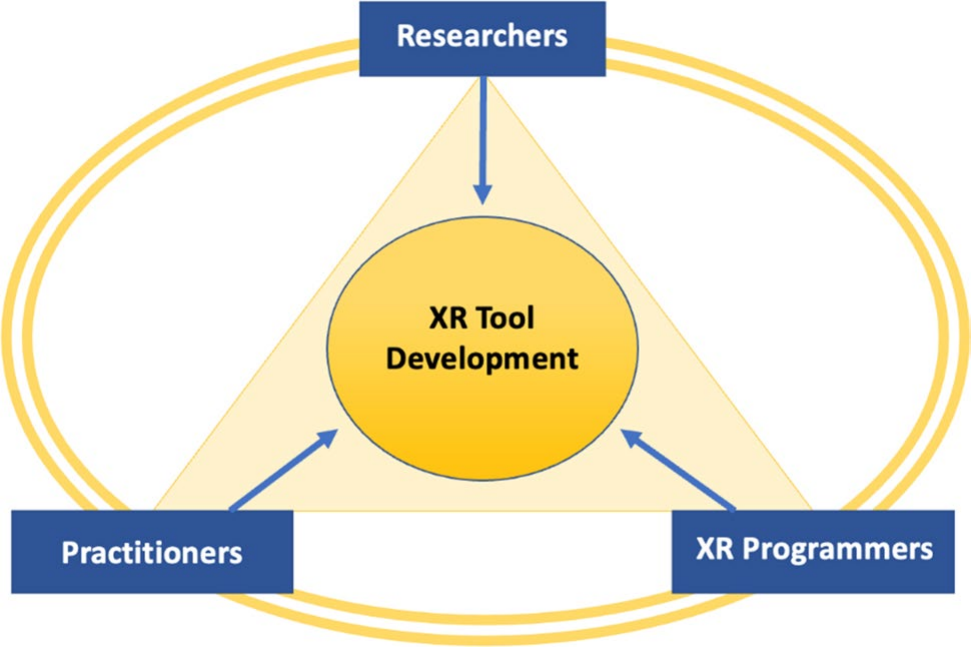
Partnership required for effective development of extended reality (XR) training tools

Finally, the effectiveness of XR within high-performance sport in the future will likely be determined by how well practitioners, researchers and XR programmers can work together (see Fig. 2). This is a key partnership whereby all parties rely upon each other’s expertise and knowledge for success. The researchers and practitioners bring to the table ideas about how they would like to use XR in sport, which are based upon research findings, practical experiences, limitations of current training methods, and skill acquisition principles or frameworks. The XR programmers have expertise in actually developing the tool, knowing the hardware and software development required to implement XR tools, and understanding the resources and costs involved. Therefore, XR programmers can inform researchers and practitioners about whether XR training ideas are possible to achieve, the length of time it will take to achieve the outcome, and the data required to create a highly representative XR tool. This is an iterative process whereby all parties use their expertise to help each other develop the most effective XR training tool [85, see also 86].

### Research Directions

The utilisation of XR within sport is certainly in its infancy and there are many unknowns surrounding this technology. Therefore, research plays a crucial role in teasing out the strengths, weaknesses and opportunities of XR and how it can be utilised to improve sports performance. Although there are many possible research prospects regarding the use of XR in sport, we highlight below what we believe to be the most critical at this early stage of XR utilisation in sport.

First, the performer-environment interactions between learners and XR tools need to be assessed with greater rigor. For example, are gaze behaviours, decisions and actions representative of those in the real-world task? Although findings from past research investigating the representativeness of movements are promising [46], assessments in the future should consider other means such as comparing gaze behaviour and movement biomechanics in the XR setting compared to the real-world setting. This is an important consideration for understanding the degree to which the perceptions and actions in the XR environment are likely to transfer to the normal performance setting and also whether such training could result in a negative skill transfer.

Second, the capability of XR tools for eliciting representative pressure responses in sport requires further investigation [87]. Future research could use a combination of physiological, subjective and transfer measures to assess the capability of XR to create representative pressure responses that promote positive adaptations. Creating pressure in training is a challenge for practitioners and coaches [11, 28] and thus the capability of XR to simulate real-world situations may provide an opportunity to practice under difficult-to-recreate competition stressors (e.g. venues, crowds and noises). Based on evidence from the field of psychology, animated VR may be most suitable for achieving these outcomes [82].

Third, researchers should take advantage of the use of artificial intelligence to manipulate contextual information and create specific scenarios. Extended reality coupled with artificial intelligence software allows researchers to manipulate any aspect of contextual information in the environment. For example, the tactics and physical abilities (i.e. speed and agility) that a virtual tennis player uses during a virtual tennis match can be controlled and/or matched with real-world tennis players (e.g. Roger Federer’s serving and groundstroke patterns of play and speed around the court). This is achieved by utilising data from players’ past performances during competition (e.g. GPS data) and using artificial intelligence to identify exactly what behaviours or movement patterns teams or individual players are using in specific situations. Therefore, this creates opportunities for researchers to train players’ perceptual-cognitive skills in specific scenarios to better prepare them for competition.

Last, future XR research should consider the inclusion of transfer tests and placebo and control groups when undertaking training interventions. A key issue with past research remains the lack of transfer tests, or monitoring of in-situ performance, to assess whether training improvements transfer to performance in competition [88]. Additionally, the majority of past research in sport has failed to include adequate placebo and control groups, as well as appropriate training intervention periods and retention tests that assess whether performance improvements are maintained over time (days, weeks, months) [89–91].

### Areas for Consideration

There is reason to question whether the normal functionality of the ventral (vision for perception) and dorsal (vision for action, specialising in visual control of skilled movement) streams are maintained when performing in the animated VR environment [92]. Importantly, the dorsal stream may be disrupted when using animated VR because of the artificial presentation of depth information [93, 94]. Research has shown that animated VR impairs the estimation of distance, and users have a perception that the VR world is flatter [94]. This causes greater reliance on monocular information and an increased use of the ventral stream to guide actions, which can cause movement inefficiency [95]. Given the dorsal stream is predominantly relied upon in the real world to guide action, there are concerns that visually guided actions performed in XR that rely predominantly on the ventral system may function using different mechanisms compared with real-world settings (for a detailed review, see Harris et al. [92]. Therefore, this may reduce the level of positive skill transfer possible from XR to the real-world environment.

Another concern is the lack of haptic feedback when interacting with virtual objects in XR environments [92]. The current haptic feedback used in XR environments is predominantly achieved through hand-held controllers, which use vibrations to signal the user has touched or made contact with a virtual object. Whilst this review has focused on the visual perceptual systems contribution to the regulation of action, it is likely the perception of other sources of information such as acoustics and haptics is equally important. Therefore, XR tools that afford non-representative haptics are likely to have the same detrimental learning and transfer effects as observed with visual information, and may cause a further reliance on the ventral stream [10]. Indeed, a recent study found that when reaching to a target in an animated VR environment where the user’s hand was represented by a cursor and no haptic feedback was present, the user’s movement kinematics indicated greater reliance on ventral pathways and weakened the user’s online corrective processes, despite visual feedback being available [96].

## Conclusions

Extended reality technology is certainly in its infancy when it comes to being utilised in sport. While there is a lack of research on the use of XR in sport, the existing findings suggest that XR may be a promising tool for sports training, particularly perceptual-cognitive skill training. However, it is important to base the design of sport-specific XR tools on the key principles of ecological dynamics and RLD, and utilise the MPTF to ensure that XR tools are highly representative of the real-world performance environment to maximise positive transfer. To validate the use of XR tools and minimise the probability of negative transfer effects, it is essential that XR tools are assessed for their level of representativeness before they are used during training [9, 10]. As interest in XR technology grows throughout the high-performance sport landscape, it is important to maintain a balanced and evidence-based approach when deciding how XR can best be utilised within training programmes.
